# Differential Response of Müller Cells and Microglia in a Mouse Retinal Detachment Model and Its Implications in Detached and Non-Detached Regions

**DOI:** 10.3390/cells10081972

**Published:** 2021-08-03

**Authors:** Seung-Hee Lee, Yong-Soo Park, Sun-Sook Paik, In-Beom Kim

**Affiliations:** 1 Department of Anatomy, College of Medicine, The Catholic University of Korea, 222 Banpo-daero, Seocho-gu, Seoul 06591, Korea; seunghui6310@daum.net (S.-H.L.); yongsoopark88@gmail.com (Y.-S.P.); paikss@catholic.ac.kr (S.-S.P.); 2 Catholic Neuroscience Institute, College of Medicine, The Catholic University of Korea, 222 Banpo-daero, Seocho-gu, Seoul 06591, Korea; 3 Department of Biomedicine & Health Sciences, Graduate School, The Catholic University of Korea, 222 Banpo-daero, Seocho-gu, Seoul 06591, Korea; 4 Catholic Institute for Applied Anatomy, College of Medicine, The Catholic University of Korea, 222 Banpo-daero, Seocho-gu, Seoul 06591, Korea

**Keywords:** GFAP, osteopontin, Müller cells, microglia, retinal detachment

## Abstract

Retinal detachment (RD) is a sight-threatening condition, leading to photoreceptor cell death; however, only a few studies provide insight into its effects on the entire retinal region. We examined the spatiotemporal changes in glial responses in a mouse RD model. In electroretinography, a- and b-waves were reduced in a time-dependent manner. Hematoxylin and eosin staining revealed a gradual decrease in the outer nuclear layer throughout the retinal region. Terminal deoxynucleotidyltransferase dUTP nick end labeling (TUNEL) assay showed that TUNEL-positive photoreceptors increased 5 days after RD and decreased by 14 days. Glial response was evaluated by immunohistochemistry using antibodies against glial fibrillary acidic protein (GFAP, Müller glial marker) and Iba-1 (microglial marker) and osteopontin (OPN, activated microglial marker). GFAP immunoreactivity increased after 7 days in complete RD, and was retained for 14 days. OPN expression increased in microglial cells 3–7 days after RD, and decreased by 14 days in the detached and border regions. Although OPN was not expressed in the intact region, morphologically activated microglial cells were observed. These retinal glial cell responses and photoreceptor degeneration in the border and intact regions suggest that the effects of RD in the border and intact retinal regions need to be understood further.

## 1. Introduction

Retinal detachment (RD) is a sight-threatening condition, in which the outer segments of the photoreceptors physically separate from the underlying retinal pigment epithelium (RPE), which acts as a photoreceptor nourishment source. Photoreceptor cell death continues during RD, causing progressive visual impairment. RD can be classified into three types: rhegmatogenous, tractional, and exudative. Rhegmatogenous detachment is the most common cause of RD, which is characterized by retinal tear-inducing vitreous penetration under the retina [1], while tractional detachment and exudative detachment are rare types of RD caused by a complication of various retinal disorders, including age-related macular degeneration (AMD) [2] and diabetic retinopathy (DR) [3].

Many studies have used various animal RD models to understand its pathogenesis [4,5]. Progressive photoreceptor cell death occurs in multiple ways in the detached region of the retina, including via apoptosis [6,7], necroptosis [8,9,10], and autophagy [11,12]. Based on the findings of microglial activation and macrophage infiltration into the subretinal space [13,14], local inflammation is thought to be involved in RD progression. However, there have been only a few studies providing an overall insight into the entire retinal region that includes intact (undetached), borderline, and detached regions.

Previously, we established and characterized animal retinal degeneration models, such as N-methyl-nitrosourea (MNU)-induced rat model [15] and a blue light-emitting diode (LED)-induced mouse model [16]. In these retinal degeneration models, we found that microglial cells and Müller glial cells were activated, and an increase in pro-inflammatory cytokines was seen [15,16,17,18,19], suggesting that inflammation is a critical mechanism in its pathogenesis. In addition, we introduced the differential activation patterns of two retinal glial cells, Müller glial cells, and microglial cells. In other words, Müller glial cells gradually express the glial fibrillary acidic protein (GFAP), an activation marker of Müller glial cells, from their foot process in the nerve fiber layer to the outer retina in the entire retina, regardless of injury location and type [15,16,17]. On the other hand, microglial cells express osteopontin (OPN), a newly identified marker for activated microglial cell marker/retinal inflammation, near the injury location, but not far from the injury region [17].

In this study, we examined the morphological characteristics of a mouse RD model with deconstructed photoreceptor outer segments and apoptotic photoreceptors [20]. In particular, we focused on retinal glia located in different regions (detached, border, and intact) of the detached retina by using three markers for retinal glial cells and inflammation: GFAP for Müller glia and retinal stress, Iba-1 for microglia, and OPN for microglia and neuroinflammation. Additionally, we tested whether OPN is a lesion-specific marker of retinal inflammation in RD, as shown in a blue LED exposure-induced retinal degeneration model [16].

## 2. Materials and Methods

### 2.1. Animals and RD Generation

All mice-related experiments were performed according to the regulations of the Catholic ethics committee of the Catholic University of Korea, Seoul, which are based on the National Institute of Health (NIH) guidelines for the Care and Use of Laboratory Animals (NIH Publication NO. 80-23), as revised in 1996. The experimental procedures were approved by the Institutional Animal Care and Use Committee of the College of Medicine, The Catholic University of Korea (approval number: CUMC-2019-0266-06).

A total of 50 male C57BL6 mice which were 6–7 weeks old were used in this study. The animals were kept in a 12-h light/12-h dark cycle in a climate-controlled laboratory. Before RD, the mice were anesthetized by intraperitoneal injection of 20 mg/kg zolazepam and 7.5 mg/kg xylazine, and the pupils were dilated using 0.5% topical tropicamide and 0.5% phenylephrine hydrochloride (Mydrin-P; Santen Pharmaceutical Co. Ltd.; Osaka, Japan). RD was induced by subretinally injecting 2 μL PBS through the sclera, by using Nanofil Application Kits (World Precision Instruments, Sarasota, FL, USA) with a 34-gauge blunt needle. This administration resulted in a detached lesion approximately 700 μm in diameter. Electroretinography (ERG) recordings were performed 1, 3, 5, 7, and 14 days after RD, and the mice were subsequently sacrificed.

### 2.2. ERG

ERG recordings were performed following the procedures described in our previous study [16]. All animals were kept in a completely dark room for 16 h before the ERG recording. All procedures were performed under dim red light (λ > 600 nm). The mice were anesthetized by intraperitoneal injection of 20 mg/kg zolazepam and 7.5 mg/kg xylazine. The corneas were coated with hydroxypropyl methylcellulose gel and covered with gold ring contact electrodes. A ground electrode and reference electrode were placed subcutaneously in the tail and ear, respectively. Stimuli were short-term white flashes, delivered via a Ganzfeld stimulator (UTAS-3000; LKC Technologies, Gaithersburg, MD, USA).

The signals were amplified and filtered through a digital band-pass filter, ranging from 5 Hz to 300 Hz to yield a- and b-waves. All scotopic ERG, rod-mediated responses were obtained at the following increasing light intensities: 0.99 and 3.96 cd/s.m^2^. Each recording was the average of all responses obtained within a 15 s inter-stimulus interval. The amplitude of the a-wave was measured from the baseline to the maximum a-wave peak, and the b-wave was measured from the maximum a-wave peak to the maximum b-wave peak.

### 2.3. Hematoxylin and Eosin (H&E) Staining

The eyecup was fixed by immersion in 4% paraformaldehyde in the 0.1 M phosphate buffer (PB; pH 7.4) for 2 h. Then, the tissue was rinsed in PB, transferred to a 30% sucrose solution in PB, infiltrated overnight, and embedded the next day in a supporting medium for frozen tissue specimens (Tissue-Tek O.C.T compound; Sakura; Alphen aan den Rijn, The Netherlands). Vertical retinal sections (8 μm in thickness) were prepared using a cryostat at −25 °C, stored at −20 °C, and then stained with H&E.

### 2.4. Terminal Deoxynucleotidyl Transferase dUTP Nick End Labeling (TUNEL) Assay

As described in our previous report [16], the TUNEL assay was performed following the manufacturer’s protocols (In Situ Cell Death Detection Kit; Roche Biochemical; Mannheim, Germany) to detect retinal cell death. In cryosections of the eyecup preparations, cell nuclei were counterstained with 4,6-diamidino-2-phenylindole (DAPI; dilution, 1:1500; Roche Biochemical). The number of TUNEL-positive photoreceptors in the outer nuclear layer (ONL) was counted in two sections of the detached, border, and intact retinal regions.

### 2.5. Immunohistochemistry

Immunohistochemical analysis was performed as described in our previous study [17]. Cryostat retinal sections of 8 μm thickness were washed three times with 0.1 M PB for 10 min each, and incubated in 10% normal donkey serum for 1 h at room temperature. The tissues were then incubated with primary antibodies, including rabbit anti-Iba-1 (dilution, 1:500; Wako Pure Chemical Industries; Osaka, Japan), mouse anti-GFAP (1:1500; Chemicon; Temecula, CA, USA), and goat anti-OPN (dilution, 1:1000; R&D Systems, Minneapolis, MN, USA) antibodies overnight at 4 °C. Then, the tissues were thoroughly rinsed in PB and incubated with appropriate secondary antibodies conjugated with Cy3 (dilution, 1:2000; Jackson ImmunoResearch; West Grove, PA, USA) or Alexa 488 (dilution, 1:1000; Molecular Probes; Eugene, OR, USA) for 2 h at room temperature. After rinsing several times in PB, the cell nuclei were counterstained with DAPI for 10 min and mounted with anti-fading mounting media (Vector Laboratories; Burlingame, CA, USA).

A Zeiss LSM 800 confocal microscope (Carl Zeiss Co. Ltd., Oberkochen, Germany) was used for observation and image acquisition. Quantitative image analysis was performed using Zen 2.3 software (Blue edition; Carl Zeiss), as described in our previous study [18]. The region of interest (ROI) was selected in the detached, border, and intact regions (480 μm length) of each retinal section, and the intensity of GFAP immunoreactivity was automatically measured.

### 2.6. Statistical Analysis

All statistical analyses for ERG amplitude, histology image analysis, TUNEL-positive quantification, and immunohistochemistry were performed using GraphPad Prism 8.0 (GraphPad Software; San Diego, CA, USA) by one-way ANOVA with Bonferroni’s multiple comparisons test. Differences were considered statistically significant at *p* < 0.05.

## 3. Results

### 3.1. Functional and Histological Changes in Experimental RD

**Figure 1:**
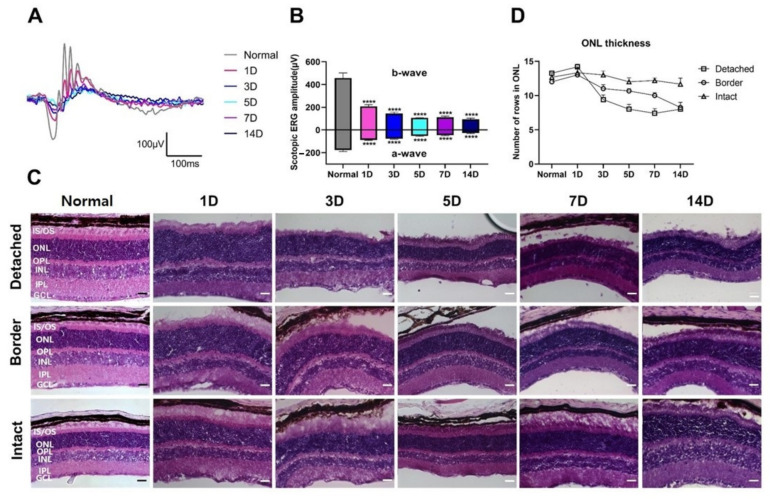
Functional and histological evaluation of retinal detachment (RD) at different time points. Electroretinograms (ERGs) were recorded in RD mice. (**A**) Representative scotopic ERG responses in normal (gray) and RD mice after 1 (pink), 3 (blue), 5 (cyan), 7 (purple), and 14 days (navy). Based on the functional changes, the amplitudes of both scotopic a- and b-waves significantly reduced in a time-dependent manner, compared to those in the normal group. (**B**) Values are represented as the mean ± SEM (*n* = 5, *p* < 0.001, one-way ANOVA). (**C**) Hematoxylin and eosin (H&E) staining of representative vertical sections from normal control and the detached retina at different time points (1, 3, 5, 7, and 14 days). Retinal thickness of the outer nuclear layer (ONL), where the photoreceptor residue gradually decreased. Scale bars, 20 μm. (**D**) ONL thickness was measured manually at each time point. According to histological changes in the detached region, ONL thickness significantly reduced in a time-dependent manner, and no significant changes were observed in the intact region. IS/OS, inner segment and outer segment; OPL, outer plexiform layer; INL, inner nuclear layer; IPL, inner plexiform layer; GCL, ganglion cell layer.

First, we evaluated the functional and histological changes in detached retinas. Functional changes in RD mice were investigated using ERG recordings. Figure 1A shows the scotopic ERG response in normal and RD mice at 1, 3, 5, 7, and 14 days after RD as representative waveforms of scotopic 0 dB flash at 0.99 cd·s/m^2^. The amplitudes of the ERG responses were significantly reduced in a time-dependent manner, compared to those in the normal group (Figure 1B, *p* < 0.001, *n* = 5 in each group). The ERG responses decreased abruptly in the early phase (within five days after RD). Afterward, the responses slightly decreased or were sustained until the last day of the experiment (14 days after RD).

Next, to assess the histology, retinal vertical sections collected from the mice after ERG recording were stained with H&E (Figure 1C). Consistent with the results of ERG, ONL thickness gradually decreased in the detached retinal regions with photoreceptor degeneration. Specifically, ONL thickness in the detached region abruptly decreased (~14 to ~7 rows of photoreceptors) in the early phase, and then remained steady (Figure 1C,D). However, ONL thickness decreased in the border and intact regions at a relatively uniform pace, although the steepness in the border region was sharp, while that in the intact region was gradual (Figure 1D).

### 3.2. Photoreceptor Degeneration in Experimental RD

**Figure 2:**
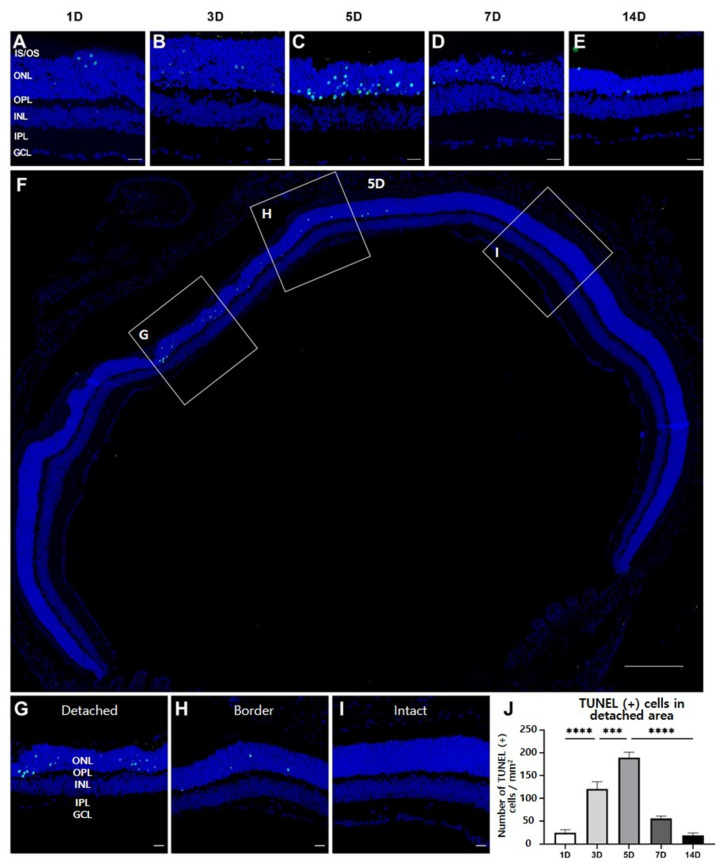
TUNEL assay to evaluate the apoptotic cell death in RD. (**A**–**E**) TUNEL-positive cells mostly observed in ONL of the detached region were significantly increased 5 days after RD, and, thereafter, decreased by 14 days. Scale bars, 20 µm. (**F**) A low-magnification view 5 days after RD. Scale bar, 200 μm. (**G**–**I**) TUNEL-positive cells located at photoreceptors were present in the (**G**,**H**) detached and border regions, but not in the (**I**) intact region. Scale bars, 20 μm. (**J**) Quantitative analysis of the number of TUNEL-positive cells was manually conducted (*n* = 5, 5 fields per time point). Data are shown as mean ± SEM. *** *p* < 0.001 and **** *p* < 0.0001 based on one-way ANOVA followed by Bonferroni’s multiple comparisons test.

We evaluated the spatiotemporal pattern of photoreceptor degeneration after RD, using the TUNEL assay at each time point. During the entire experimental period, TUNEL-positive cells were exclusively observed in the ONL, indicating that they corresponded to photoreceptors (Figure 2A).

As expected from the histological findings shown in Figure 1C,D, most TUNEL-positive photoreceptors were observed in the detached region in the retinas (Figure 2A–C). They occurred at day 1, reached a peak at day 5, and rarely existed 14 days after RD. However, fewer TUNEL-positive photoreceptors were found in the border retinal region than in the detached retinal region, and only a few TUNEL-positive cells were found in the intact region 5 days after RD (Figure 2F–I). Thereafter, TUNEL-positive cells were manually counted at different time points (Figure 2J). TUNEL-positive cells gradually increased until 5 days after RD, after which they significantly decreased (*p* < 0.05). Together with histological results, these results demonstrate that the degeneration of photoreceptors occurs in early RD, mainly in the detached region, while progressing slowly in the border and intact regions.

### 3.3. GFAP Expression in Müller Glial Cells in RD

To assess retinal injury or stress in RD, immunohistochemical assays with anti-GFAP, a widely used marker for retinal injury and/or reactive Müller glial cells [15,17,21], were performed.

**Figure 3:**
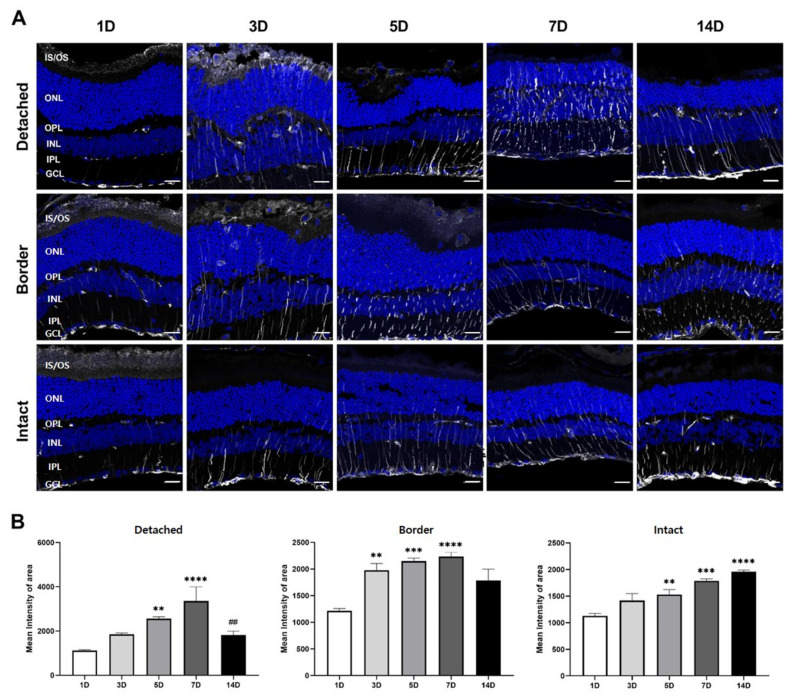
GFAP expression in mouse detached retinas. (**A**) Confocal micrographs of vertical cryosections collected 1, 3, 5, 7, and 14 days after RD. The sections were immunostained with anti-GFAP (white), an activated Müller glial cell marker. In the detached region, GFAP immunoreactivity gradually increased until 7 days after RD, and then decreased. In the border and intact regions, GFAP expression was significantly weak. Scale bars, 20 μm. (**B**) In each region, ROIs were positioned manually per outer nuclear layer (ONL; *n* = 6, 15 fields per time point). In the detached region, GFAP expression gradually increased until 7 days after RD (*p* < 0.05), after which it significantly decreased (*p* < 0.05). In border regions, GFAP levels tended to increase or at least be sustained by 14 days after RD. In the intact region, GFAP expression steadily increased time-dependently (*p* < 0.05). Data are shown as mean ± SEM. ** *p* < 0.01, *** *p* < 0.001, and **** *p* < 0.0001 versus 1 day after RD, and ^##^ *p* < 0.01 versus 7 days after RD, based on one-way ANOVA followed by Bonferroni’s multiple comparisons test.

As shown in Figure 3A, GFAP immunoreactivity was observed in the inner limiting membrane and in few thin Müller cell processes in the inner plexiform layer (IPL) and inner nuclear layer (INL) of the detached retinal region, as well as in border and intact regions at 1 day after RD, similar to a normal retina (data not shown). However, GFAP immunoreactivity was observed in many Müller cell processes in the IPL, INL, outer plexiform layer (OPL), and ONL in the entire retina 3 days after RD. GFAP expression peaked 7 days after RD in the detached region, where many GFAP-immunoreactive Müller cell processes were distributed throughout all retinal layers, and some even appeared stout. In contrast, GFAP expression was significantly reduced in the border and intact regions, compared to that in the detached region. GFAP immunoreactivity in the detached region decreased 14 days after RD. However, GFAP immunoreactivity in the border region was similar to that at 7 days after RD, while that in the intact region sometimes appeared to be stronger than that at 7 days after RD.

The images captured after immunohistochemical analysis for GFAP were quantified as the GFAP profile per ONL, where photoreceptors degenerate (Figure 3B). Quantification results demonstrated that GFAP expression in the detached region gradually increased until 7 days after RD, after which it significantly decreased (*p* < 0.05). Moreover, GFAP expression slightly increased or was sustained in both the border and intact regions 14 days after RD. These results suggest that GFAP expression may be a general, but not a lesion-specific, marker for detecting retinal injury or stress in RD.

### 3.4. OPN Expression in Iba-1-Immunolabeled Microglial Cell in RD

We also evaluated retinal inflammation, and, thus, performed double-labeling immunofluorescence with anti-Iba-1, a microglial cell marker [17,22,23], and anti-OPN, an activated microglial cell marker [24,25,26], which was used for a new marker for retinal injury [17,27].

**Figure 4:**
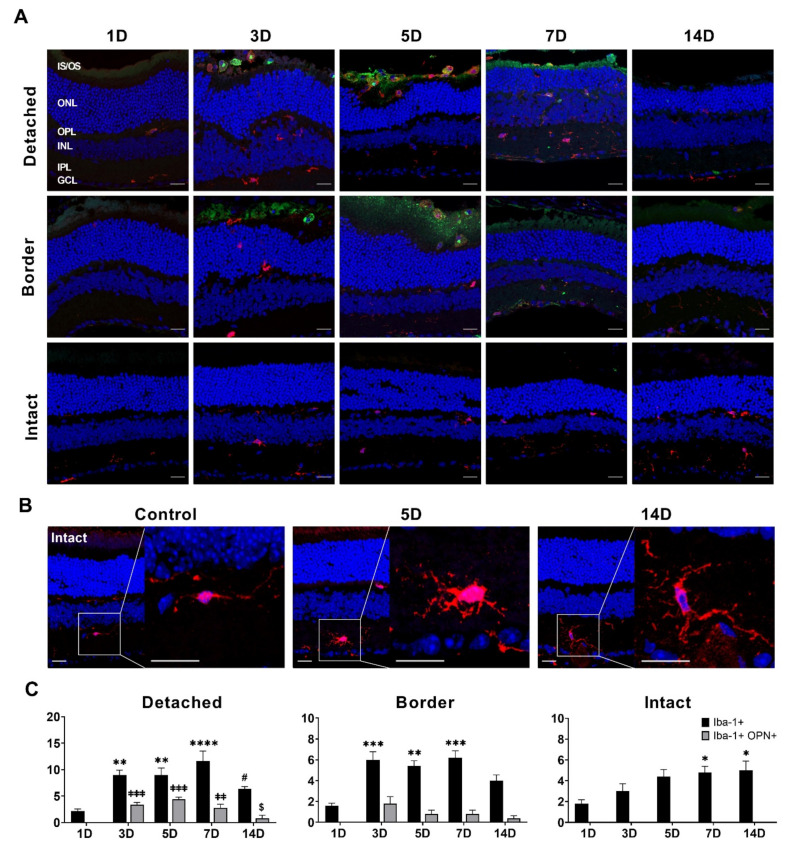
Iba-1 and OPN expression in mouse RD model. (**A**) Confocal micrographs of vertical cryosections collected 1, 3, 5, 7, and 14 days after RD. The sections were double immunostained with anti-Iba-1 (red) as a microglial cell marker and anti-OPN (green) as an inflammation marker. Iba-1 and OPN co-labeled microglial cells were mainly detected from 3 to 7 days after RD, and, thereafter, decreased by 14 days after RD in the detached and border regions. In the intact region, the number of Iba-1-labeled microglial cells was lower than that in the detached region, and OPN co-labeled microglial cells were undetected. OPN immunoreactivity was detected only in the subretinal space, but Iba-1 immunoreactivity was observed in all the layers. Scale bars, 20 μm. (**B**) Representative Iba-1-labeled microglial cells at the normal control, peak, and end of the experiment in the intact region. Iba-1-labeled microglial cells showed changes in their morphological characteristics. Scale bars, 20 μm. (**C**) Quantitative analysis of the number of Iba-1 and OPN co-labeled cells was conducted. In each region, the number of Iba-1- and OPN co-labeled cells were measured for all layers. Iba-1-labeled, and not OPN co-labeled, microglial cells were observed in each region. Most OPN co-labeled microglial cells were detected in the detached and border regions, but not in the intact region (*n* = 6, 15 fields per time point). Data are shown as mean ± SEM. * *p* < 0.05, ** *p* < 0.01, *** *p* < 0.001, **** *p* < 0.0001, ^ǂǂ^ *p* < 0.01 and ^ǂǂǂ^ *p* < 0.001 versus 1 day after RD, and ^#^ *p* < 0.05 and ^$^ *p* < 0.05 versus 7 days after RD based on one-way ANOVA followed by Bonferroni’s multiple comparisons test.

Few Iba-1-labeled microglial cells were found in the OPL and IPL of the entire retinal regions, including detached, border, and intact (normal data not shown) regions at 1 day after RD, and OPN immunoreactivity was rarely observed in normal retinas at 1 day after RD (Figure 4A). The number of Iba-1-labeled microglial cells was significantly increased in the detached and border regions, but not in the intact regions (*p* < 0.05) 3 days after RD. In the detached and border regions, Iba-1-labeled microglial cells were found throughout the retinal layer from the IPL to the subretinal space. OPN immunoreactivity was observed in Iba-1-labeled microglial cells in the subretinal space, but not in the retina. The number of Iba-1-labeled and OPN co-labeled microglial cells increased in both detached and border regions 5 days after RD. In addition, OPN immunoreactivity was observed in the rod and cone layers, which may be fragmented or degenerating photoreceptor outer and inner segments, as previously reported [17]. The number of Iba-1-labeled microglial cells in the detached and border regions peaked 7 days after RD. Interestingly, Iba-1-labeled microglial cells decreased in the subretinal space, while those within the outer and inner retina increased. The number of OPN co-labeled microglial cells decreased because of the decrease in Iba-1-labeled microglial cells in the subretinal space. The number of Iba-1-labeled microglial cells markedly decreased, particularly in the subretinal space, 14 days after RD; thus, OPN co-labeled microglial cells were rarely observed. In the intact region, throughout the entire experimental period, Iba-1-labeled microglial cells were only observed within the retina but not in the subretinal space; thus, OPN immunoreactivity was not observed. Iba-1-labeled microglial cells within the retina slightly increased in number and changed in size and shape. That is, they were stout and their processes were thick and long compared to the Iba-1-labeled microglial cells in the normal retina (Figure 4B).

The images captured after immunohistochemical analysis for Iba-1 and OPN were measured for all of the layers (Figure 4C). In the detached region, Iba-1-labeled cells significantly increased until 7 days after RD (*p* < 0.05) and decreased by 14 days after RD in the detached and border regions (*p* < 0.05). OPN co-labeled microglial cells were detected from 3 days after RD and were rarely observed 14 days after RD. In the intact region, the number of Iba-1-labeled cells increased from 3 to 7 days after RD, and then sustained for 14 days, unlike those in detached and border regions. OPN co-labeled microglial cells were not observed during the experimental period.

## 4. Discussion

RD is an eye disorder in which photoreceptors separate from the choroidal blood vessels that provide oxygen and nourishment. It can occur spontaneously as a complication from various retinal disorders, such as AMD and DR. Untreated RD can lead to permanent vision loss in the affected eye. Nevertheless, RD pathogenesis is little known, compared to other retinal diseases, including AMD and DR. In particular, events that occur in non-detached areas, including the penumbra, have not been extensively studied. This might be because surgery is almost always performed for the treatment of RD, and its outcome is generally good [28].

The study demonstrated progressive photoreceptor loss in the detached region with a decreased ERG response (Figure 1). TUNEL-positive cells were first observed in the detached region 1 day after RD, prominently increased from 3 days after RD, and peaked at 5 days after RD (Figure 2). These findings reveal that photoreceptor degeneration starts early (<24 h), accelerates, and rapidly subsides within a week, suggesting that surgical intervention is recommended in early RD [29]. In addition, we observed an indispensable number of TUNEL-positive cells in the border region near the detached region, while histological findings showed thinned ONL. Additionally, in a few cases, the ONL thickness in the intact region decreased, albeit statistically insignificant (*p* > 0.05). Inflammation in the border region was comparable to that in the detached region. These findings also suggest that early intervention in RD is important for preventing its progression into the neighboring regions of the retina.

Many studies have been conducted in order to understand the consequences of induced RD in animal models, and have strongly implicated inflammatory factors [13,14,30,31,32]. Two retinal glial cells, Müller glia and microglia, play a key role in the inflammatory response in the retina [33,34]. Thus, characterizing the glial response activation pattern is important for interpreting the inflammatory processes. Previously, we reported the differential activation patterns of two retinal glial cells, Müller glial cells and microglial cells, in two different RD models. Müller glial cells were gradually activated and slowly subsided in whole retinal regions, regardless of the injury site and type [16,17], whereas microglial cells were abruptly activated and deactivated at the injury site, but not far from the injury region [17]. However, there have been few reports of glial responses throughout the retina in the RD model.

In this study, GFAP expression, the activation marker of Müller glial cells, increased from the inner limiting membrane of the detached region to the ONL until 7 days after RD, and then decreased slightly. GFAP expression increased similarly in the entire retina and was not limited to the detached region (Figure 3). This GFAP expression pattern has been previously reported in light-induced photoreceptor degeneration [16,35] and RD models [36,37]. Interestingly, GFAP expression increased in the intact region, regardless of RD. This finding was also reported in our previous studies in the blue LED-induced retinal degeneration model [16,17]. Taken together, these findings suggest that regardless of the injury size and type, Müller cells in the entire retina respond to injury.

Microglial distribution was more distinct than Müller glial distribution in RD. Iba-1-labeled microglial cells were detected in the subretinal space and ONL in the detached and border regions from 3 to 14 days after RD, with a peak at 7 days, but not in the intact region. Interestingly, Iba-1-labeled microglial cells in the subretinal space peaked 5 days after RD and showed OPN immunoreactivity (Figure 4), and also expressed major histocompatibility class II (MHC-II) (Supplementary Figure S1). This is consistent with previous studies, in which MHC-II-positive microglial cells were located in the subretinal space in the retinal degeneration model, suggesting that they play a pro-inflammatory and phagocytic role during photoreceptor degeneration in a region-specific manner [38,39]. In addition, they express OPN, which is secreted by microglia and acts as a pro-inflammatory cytokine [24,25,26,40]. In contrast, within the retina, Iba-1-labeled microglial cells peaked 7 days after RD, followed by a slight decrease at 14 days after RD in the detached region. Moreover, they tended to proliferate in the intact region. In addition, some Iba-1-labeled microglial cells showed activated microglial morphology characterized by a stout cell body and thick and extended processes in the intact region at 14 days after RD, compared to those in normal retinas (Figure 4B). These findings suggest that microglial cells can be activated by the propagation of alarm signals from the injury site, and are involved in systemic inflammation in the retina. This inference is supported by previous studies, showing that unilateral ocular hypertension and axotomy induce contralateral microglial cell activation (e.g., the advent of microglial cells with activated form, MHC-II upregulation, and increase in Iba-1-labeled microglial cell number) [41] and retinal neuronal degeneration in the contralateral eye [42]. Taken together, inflammation during RD in the detached region could be propagated to the intact region, eventually leading to photoreceptor degeneration. Further studies are needed to elucidate the signaling molecules that mediate alarm signals from detached subretinal microglial cells to these cells in the intact region.

Lastly, this study aimed to confirm that OPN is a good lesion-specific marker for retinal inflammation in RD. OPN is upregulated or secreted by activated microglial cells in various types of brain injury [43,44,45,46] and RD [16,47]. Our findings demonstrated that OPN is expressed in activated microglial cells in the detached and border regions, but not in the intact region. Moreover, the temporal profile of OPN expression in microglial cells is more closely related to photoreceptor degeneration in RD than GFAP. This spatiotemporal correlation between OPN expression and cell death in retinal lesions has been previously reported in RD [17] and glaucoma [27]. Therefore, OPN can be used as a marker of retinal inflammation, which is accompanied by retinal injury.

## Data Availability

Data is contained within the article or supplementary material.

## Supplementary Materials

The following are available online at https://www.mdpi.com/article/10.3390/cells10081972/s1, Figure S1: MHC-II expression in a mouse RD model.
