# Supplementary material for: Differential Response of Müller Cells and Microglia in a Mouse Retinal Detachment Model and Its Implications in Detached and Non-Detached Regions
Source: Cells. 2021 Aug 3;10(8):1972. doi: 10.3390/cells10081972 (PMC8394779; doi:10.3390/cells10081972)
Supplement: Supplementary file 1 [file cells-10-01972-s001.zip › cells-1277528-supplementary.pdf]

Supplementary Figure

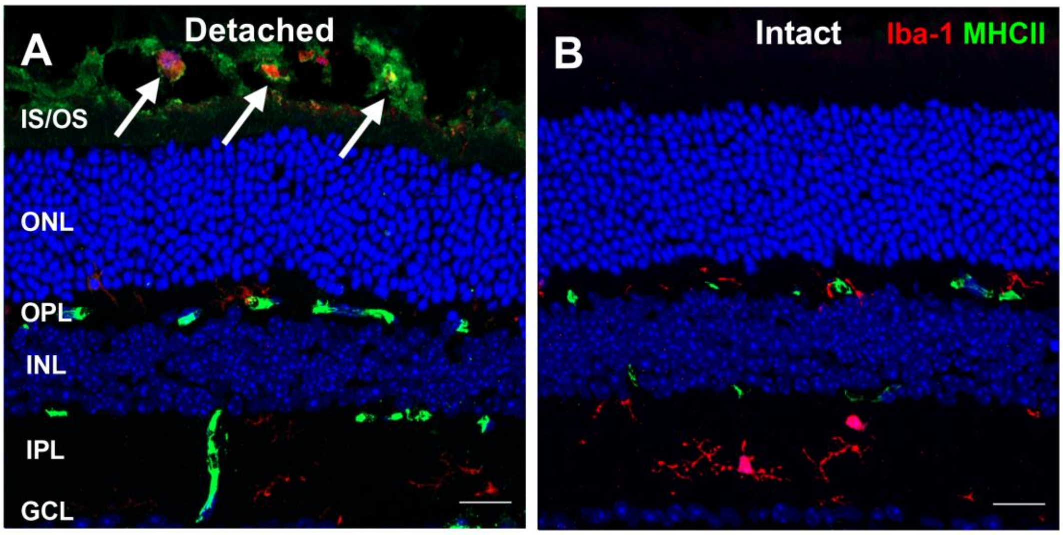

Supplementary Figure S1. MHC-II expression in a mouse RD model. (**A**,**B**) Confocal micrographs of a vertical cryosection of the retina at 5 days after RD in detached (**A**) and intact (**B**) regions. Subretinal microglial cells (arrows) in detached retina expressed MHC-II in the detached region (**A**), but not in the intact region (**B**). Scale bars, 20 μm.
